# Fluorescence lifetime DNA-PAINT for multiplexed super-resolution imaging of cells

**DOI:** 10.1038/s42003-021-02976-4

**Published:** 2022-01-11

**Authors:** Nazar Oleksiievets, Yelena Sargsyan, Jan Christoph Thiele, Nikolaos Mougios, Shama Sograte-Idrissi, Oleksii Nevskyi, Ingo Gregor, Felipe Opazo, Sven Thoms, Jörg Enderlein, Roman Tsukanov

**Affiliations:** 1 III. Institute of Physics - Biophysics, Georg August University, 37077 Göttingen, Germany; 2 Department of Child and Adolescent Health, University Medical Center Göttingen, 37073 Göttingen, Germany; 3 Institute of Neuro- and Sensory Physiology, University Medical Center Göttingen, 37073 Göttingen, Germany; 4 Center for Biostructural Imaging of Neurodegeneration (BIN), University Medical Center Göttingen, 37075 Göttingen, Germany; 5 NanoTag Biotechnologies GmbH, 37079 Göttingen, Germany; 6 Biochemistry and Molecular Medicine, Medical School, Bielefeld University, 33615 Bielefeld, Germany; 7 Cluster of Excellence “Multiscale Bioimaging: from Molecular Machines to Networks of Excitable Cells” (MBExC), Georg August University, Göttingen, Germany

**Keywords:** Super-resolution microscopy, Fluorescence imaging

## Abstract

DNA point accumulation for imaging in nanoscale topography (DNA-PAINT) is a powerful super-resolution technique highly suitable for multi-target (multiplexing) bio-imaging. However, multiplexed imaging of cells is still challenging due to the dense and sticky environment inside a cell. Here, we combine fluorescence lifetime imaging microscopy (FLIM) with DNA-PAINT and use the lifetime information as a multiplexing parameter for targets identification. In contrast to Exchange-PAINT, fluorescence lifetime PAINT (FL-PAINT) can image multiple targets simultaneously and does not require any fluid exchange, thus leaving the sample undisturbed and making the use of flow chambers/microfluidic systems unnecessary. We demonstrate the potential of FL-PAINT by simultaneous imaging of up to three targets in a cell using both wide-field FLIM and 3D time-resolved confocal laser scanning microscopy (CLSM). FL-PAINT can be readily combined with other existing techniques of multiplexed imaging and is therefore a perfect candidate for high-throughput multi-target bio-imaging.

## Abstract (web summary)

Oleksiievets et al. combine single-molecule localization super-resolution microscopy technique DNA-PAINT with fluorescence lifetime imaging to allow fast multi-target super-resolution imaging in one spectral region by separating two or three different targets by fluorescence lifetime of the associated dyes. The method is demonstrated on two complementary experimental techniques, a standard confocal laser scanning setup, and a widefield microscope employing a novel TCSPC camera as the detector.

## Introduction

Fluorescence lifetime imaging microscopy (FLIM) has become an important tool in bioimaging as it adds the fluorescence lifetime dimension to conventional intensity-based imaging. A recently introduced new generation of commercially available lifetime cameras^1,2^ features single-molecule sensitivity, making the combination of wide-field FLIM and single-molecule localization microscopy (SMLM) feasible^3^. Moreover, a recently reported variant of SMLM that employs confocal laser-scanning microscopy (CLSM) for imaging could successfully realize fluorescence lifetime SMLM (FL-SMLM)^4^. DNA-PAINT is a rapidly developing SMLM super-resolution technique capable of generating images with nearly molecular resolution^5–7^. The fact that DNA-PAINT can be readily realized with almost all conventional fluorescence microscopy modalities makes it particularly attractive. Especially interesting are DNA-PAINT combinations with CLSM, using fast scanner-based confocal microscope^4^ and with spinning disk confocal microscopy^8^. DNA-PAINT exploits several of the unique properties of DNA: stability, orthogonality, and designability. Such combination makes DNA-PAINT a favored choice for multiplexed super-resolution imaging^9–11^. In particular, bioimaging benefits from multiplexed super-resolution imaging, as it allows for co-localizing different intracellular targets with high resolution. Such information sheds light on the mutual organization, interaction, and function of different organelles^12,13^. Several experimental techniques for multiplexed imaging have been reported^14–19^, among them methods that are based on sequential label exchange using microfluidic platforms^13,20,21^. The idea behind multiplexed DNA-PAINT imaging is to reversibly bind short pieces of single-stranded DNA (imager strands) with different sequences to complementary docking strands, which are in turn attached to specific targets of interest. Among the existing multiplexed DNA-PAINT strategies, two techniques stand out. The first one, Exchange-PAINT^9,11^, performs sequential imaging of targets by introducing, one-by-one, different imager strands directed against different targets. Exchange-PAINT is capable of imaging, in principle, an unlimited number of targets. However, the total acquisition time scales linearly with the number of imaged targets and can become exceedingly long for multi-target imaging. The second technique, kinetic barcoding^22,23^, is based on the engineered on- and off-rates of imager binding kinetics. However, kinetic barcoding DNA-PAINT for multiplexed DNA-PAINT imaging of cells suffers from high variability in the binding kinetics due to the dense and sticky environment inside the cell, limiting the number of targets that can be distinguished. As an alternative, FL-SMLM enables robust identification of different species based on their fluorescence lifetimes. Here, we introduce FL-PAINT, a technique that is ideally suited for multiplexed super-resolution imaging.

## Results and discussion

### Selection of fluorophores in an orange spectral range suitable for FL-PAINT

In FL-PAINT, we distinguish between targets based on the different fluorescence lifetimes of the fluorophores conjugated to the imager strands. This requires careful selection of fluorophore candidates with sufficiently distinct lifetimes. In the present paper, we have chosen dyes emitting in the orange spectral range due to the higher quantum yield of the employed lifetime camera in this spectral region, as compared to far-red dyes used in our previous work^3^. Besides the fluorescence lifetimes of the fluorophores, additional selection criteria were brightness, reduced unspecific binding of imagers inside cells, and width of the lifetime distribution of the fluorophores. The finally selected candidates were Alexa 555, Cy3b, and Atto 550, with lifetimes of 1.7, 2.8, and 3.7 ns correspondingly (when attached to DNA), as they perfectly match the above criteria. A complete compilation of all fluorescence lifetime values and average numbers of photons emitted during a single imager-docking binding event for all fluorophore-imager combinations used in our paper is given in Supplementary Table 1.

### Wide-field FL-PAINT

**Fig. 1:**
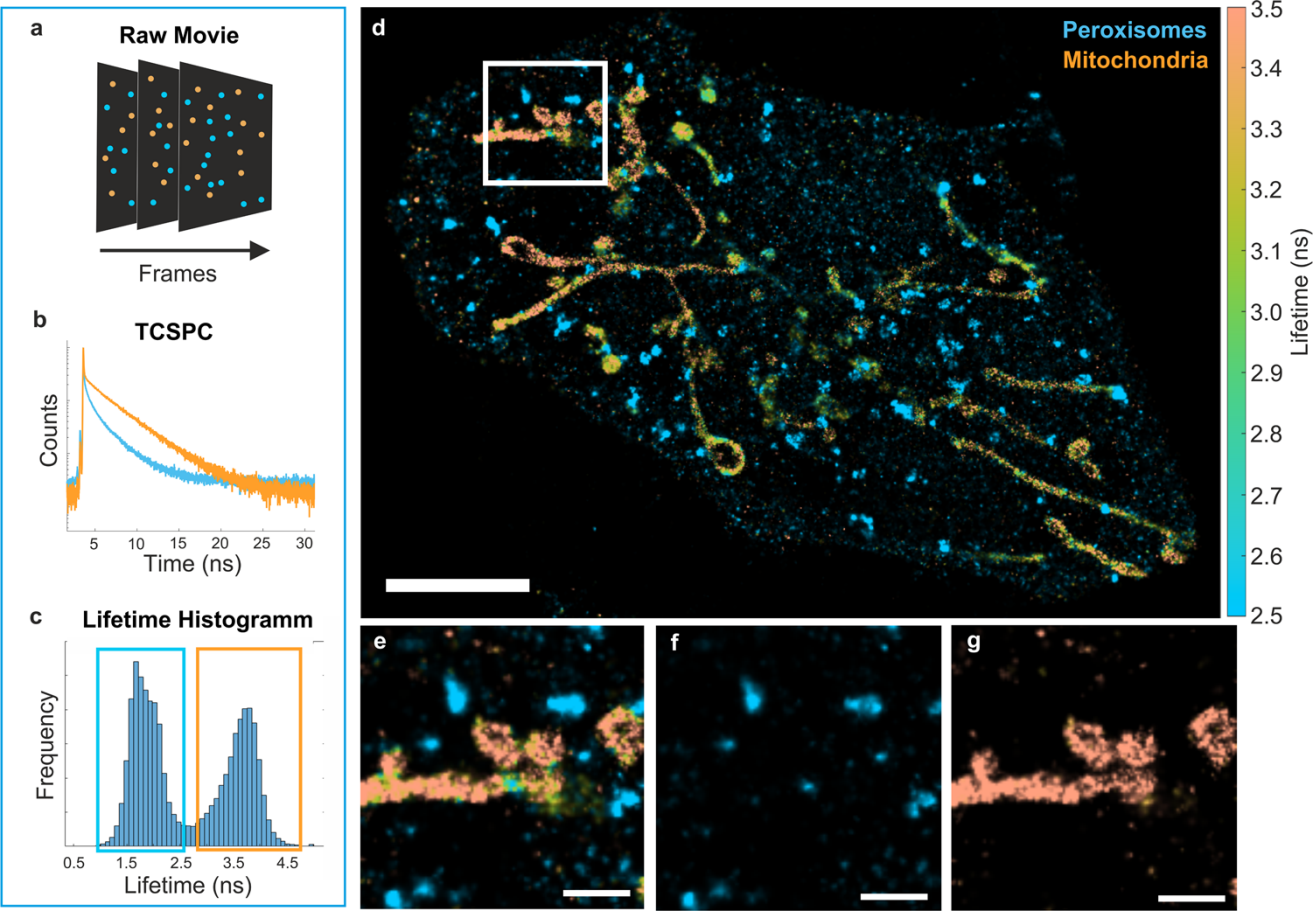
Multiplexed FL-PAINT imaging of a fixed cell. **a** Stack of DNA-PAINT frames with single-molecule localizations. Alexa 555 is depicted in blue color and Atto 550 in orange color. **b** TCSPC curves of the two fluorophores were obtained by adding all single-molecule localizations of the same type. The colors correspond to the colors used in the single-molecule image in (**a**). **c** Lifetime histogram of two-target FL-PAINT of a HeLa cell. The combination of imagers P1-Alexa 555 and P3-Atto 550 allowed for a remarkably good separation between the lifetimes of the two targets, indicated by blue and orange rectangles. **d** Reconstructed FL-PAINT image of a HeLa cell. The lifetime color bar is shown on the right-hand side of the FL-PAINT image. Scale bar is 5 µm. **e** Magnification of the white frame in the primary image. A lifetime threshold was used to separate the targets into two images: peroxisomes (**f**) and mitochondria (**g**). Scale bars are 1 µm.

In contrast to CLSM-based FLIM, wide-field FLIM is capable of fast acquisition of relatively large fields of view. Moreover, it can be combined with both total internal reflection fluorescence (TIRF)^24^ as well as highly inclined and laminated optical sheet (HILO) excitation. Recent advances in the performance of photo-multiplier tubes led to the development of a new commercial lifetime camera (LINCam, Photonscore) with large detector diameter (25 mm) and extremely high signal-to-noise ratio^25^. We have recently demonstrated that this camera is capable of detecting single molecules, even in the far-red spectral range where its quantum yield (QY) of detection is only about 2%. We were able to identify individual surface-immobilized fluorophores in a mixture of three different dyes with similar emission spectra, based solely on the fluorescence lifetime^3^. This made the combination of FLIM with SMLM possible. In the current paper, we switched to fluorophores in the orange spectral region where the camera’s QY is around 10%. For DNA-PAINT, we used imager and docking DNA sequences originally introduced by the Jungmann group^6^, which became the gold standard in the DNA-PAINT community. We also kept the original imager and docking nomenclature: imager strands P1, P2, and P3; complementary docking strands P1*, P2*, and P3*. To decrease the distance between a reporter fluorophore and a target (so called “linkage error”), we used single-domain antibodies (nanobodies), which targeted fluorescent proteins^11,26^, see “Methods” section for details. As a proof-of-concept experiment for FL-PAINT, we used a custom-built optical setup (see details in Supplementary Fig. 1a) to image two-target fixed HeLa cells: the first target being peroxisomes labeled with P1*, and the second target being mitochondria labeled with P3*, see Fig. 1. When reconstructing a super-resolution image, the arrival times of the photons were used to build time-correlated single photon counting (TCSPC)^27^ histograms for each imager-docking binding event and to obtain its lifetime value, see Fig. 1b. The resulting lifetime histogram (Fig. 1c) shows peaks at values corresponding to the different fluorophores conjugated to the imagers: P1-Alexa 555 and P3-Atto 550 for peroxisomes and mitochondria, respectively, see Fig. 1d for a whole cell image, Fig. 1e for a zoom-in, and Fig. 1f, g for each separate target. We independently verified the labeling specificity by imaging genetically encoded fluorescent proteins, see Supplementary Fig. 2. The exceptionally good separation between the peaks in the lifetime histogram allows for a straightforward target identification with a negligible crosstalk of less than 1%. The details of the crosstalk estimation are provided in Supplementary Fig. 3. The average localization precision for the image shown in Fig. 1d is 17.6 nm, the average resolution is 66.4 nm, and the minimum resolution is 21.3 nm. These values are comparable to the previously reported values using conventional Exchange-PAINT imaging^11^. For convenience, we list the values for localization presicion and resolution of image shown in Fig. 1 and all other images presented in Supplementary Table 2. In addition, we show the resolution map for image shown in Fig. 1 in Supplementary Fig. 4.

**Fig. 2:**
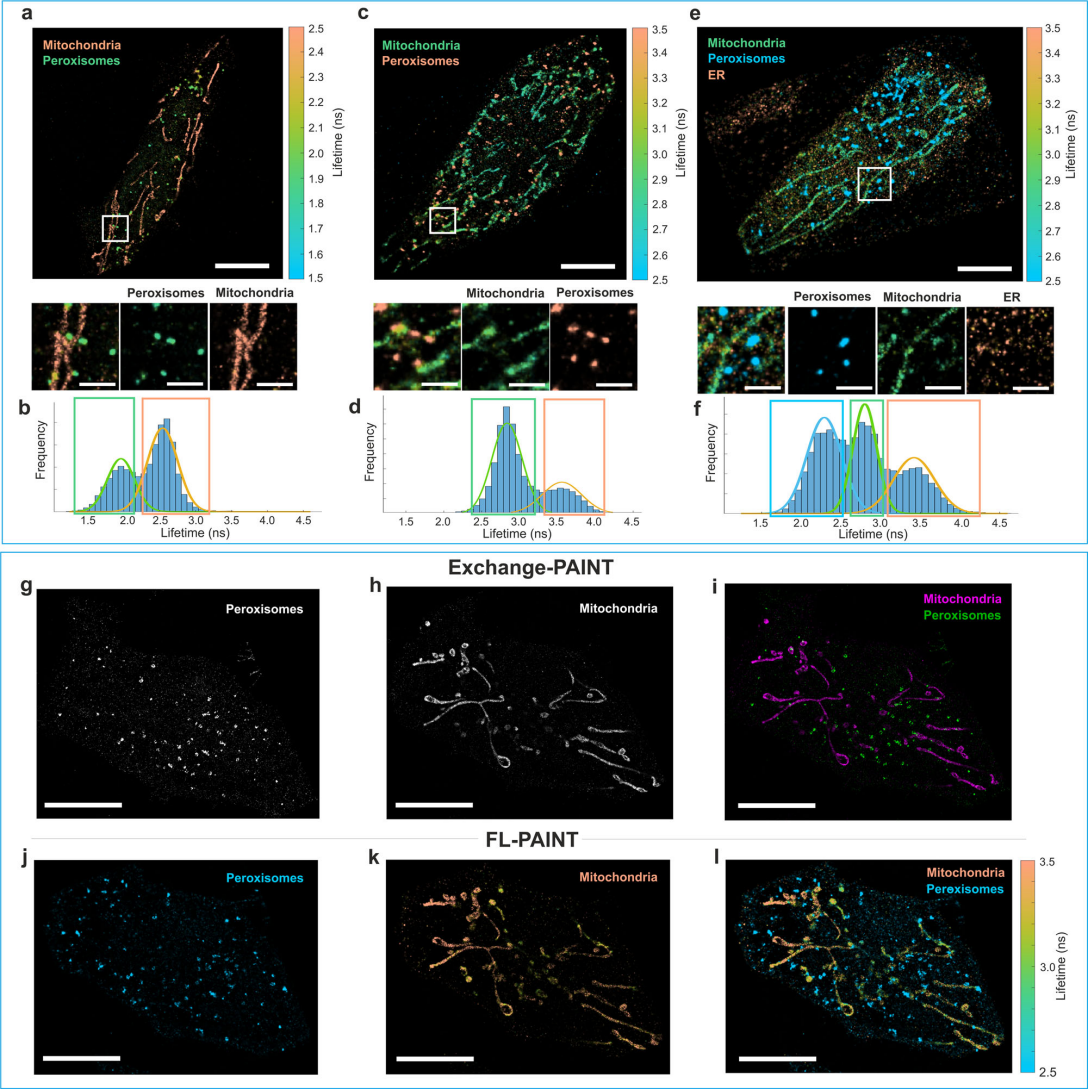
Two- and three-target FL-PAINT of HeLa cells using wide-field FLIM. **a** Two-target FL-PAINT image: peroxisomes and mitochondria labeled with imagers P1-Alexa 555 and P3-Cy3b. **b** Lifetime histogram with two Gaussian fits for the HeLa cell shown in (**a**). The two peaks in the lifetime histogram correspond to the two targets: mitochondria and peroxisomes. **c** Two-target FL-PAINT image: peroxisomes and mitochondria labeled with the two imagers P1-Atto 550 and P3-Cy3b. **d** Lifetime histogram with two Gaussian fits for the HeLa cell shown in (**c**). **e** Three-target FL-PAINT image: peroxisomes, endoplasmic reticulum, and mitochondria labeled with the three imagers P1-Alexa 555, P2-Atto 550, and P3-Cy3b, respectively. Bottom: zoom-in of white frames in the primary images with separation into different targets. Zoom-in scale bars are 2 µm. **f** Lifetime histogram with three Gaussian fits for the FL-PAINT image shown in (**e**). The blue, green, and orange rectangles show the two or three lifetime ranges associated with the different targets. A lifetime colorbar is shown on the right-hand side of the lifetime images. Scale bars are 10 µm. **g**–**l** Validating FL-PAINT with Exchange-PAINT. Exchange-PAINT images of HeLa cell with two targets: peroxisomes (**g**) and mitochondria (**h**). **i** Overlay of the two DNA-PAINT images. **j**–**l** FL-PAINT image of the same HeLa cell with two targets. **j** Reconstructed single-target image of peroxisomes. **k** Reconstructed single-target image of mitochondria. **l** Combined two-target FL-PAINT image. Scale bars in (**g**–**l**) are 5 µm.

To optimize FL-PAINT further, we examined the imaging quality for different combinations of fluorophores conjugated to imager strands. For this purpose, we performed two-target FL-PAINT imaging of HeLa cells, with the following imager-fluorophore combinations: P1-Alexa 555/P3-Cy3b (see Fig. 2a), and P1-Atto 550/P3-Cy3b (Fig. 2c). Imager P1 revealed peroxisomes, while imager P3 revealed mitochondria. To identify the targets, the resulting lifetimes were histogrammed as shown in Fig. 2b, d. In both cases, the histograms had two prominent and well-separated peaks, making lifetime-based target identification straightforward. In the next step, we performed three-target imaging of HeLa cells (Fig. 2e, f). In this case, imagers P1-Alexa 555, P2-Atto 550, and P3-Cy3b were used to target peroxisomes, endoplasmic reticulum, and mitochondria, respectively. Detailed information on the possible combinations of imagers with different fluorophores and the obtained average lifetimes, localization precisions, resolutions, and the number of localization events for each target are summarized in Supplementary Table 2. We further demonstrate the resolving power of FL-PAINT by showing the resolution map in Supplementary Fig. 4, and cross-sections of peroxisomes in Supplementary Fig. 5. Peroxisomes, due to their small dimensions (100–300 nm)^28,29^, are unresolvable for diffraction-limited microscopy and therefore are a suitable target for the demonstration of the resolving power of super-resolution techniques. The crosstalk between the targets for these images was between 2.2 and 5.7%, see Supplementary Fig. 3. A collection of additional FL-PAINT images of HeLa and COS-7 cells are shown in Supplementary Fig. 6. The quality of the separate single-target images generated from the FL-PAINT image can be further improved by applying a Bayesian pattern matching algorithm. For this purpose, reference measurements with single targets were acquired to obtain reference TCSPC curves for each target separately. The pattern matching algorithm was then applied to the mixed target data, assigning target probabilities for each binding event. Further details and processed images are presented in Supplementary Fig. 7. As pattern matching requires additional reference measurements and further data analysis, it is reasonable to use it only for particularly challenging cases, in terms of target separation.

In FL-PAINT imaging, two counteracting requirements have to be reconciled. On one hand, maximizing excitation intensity will be advantageous for increasing signal-to-noise ratio and ideally bleaching a fluorophore until unbinding. On the other hand, photon flux rates hitting the MCP-based camera (LINCam) should not exceed several hundreds kcounts per second, beyond which the camera’s temporal response becomes increasingly non-linear and the detector can even be damaged. We found that the optimal detection count rate is ~300 kcounts/s, see Supplementary Fig. 8. Therefore, different neutral density (ND) filters were used in the emission path, as listed in the Supplementary Table 2. We further investigated the impact of limiting the emission photon flux on image resolution and discovered that using an ND filter with optical density (OD) 0.3 has only a minimal impact on the localization precision; see Supplementary Figs. 9 and 10.

### Wide-field FL-PAINT validation with Exchange-PAINT

Next, we validated FL-PAINT against Exchange-PAINT that is commonly used for multiplexed DNA-PAINT imaging. For this purpose, we used HeLa cells with two labeled targets, see Fig. 2g–l. For both Exchange-PAINT and FL-PAINT, exactly the same imagers were used: P1-Atto 550 (targeting mitochondria) and P3-Cy3b (targeting peroxisomes), at a concentration of 0.5 nM for each imager. First, we performed Exchange-PAINT imaging with two imaging cycles: a first cycle for imaging peroxisomes (Fig. 2g) followed by thorough washing and then a second cycle for imaging mitochondria (Fig. 2h). For solution exchange, a custom-built microfluidic system was employed^11^. An overlay of the two images is shown in Fig. 2i. Next, we performed FL-PAINT imaging of the same HeLa cell. The sample chamber was filled with a mixture of the two imagers, the same as used in Exchange-PAINT but with lower concentration (0.1 nM for each imager). The emission light was redirected from the emCCD camera to the lifetime camera. The total acquisition time of the two-target Exchange-PAINT experiment was 70 min, close to two-fold longer than the FL-PAINT experiment (40 min). The resulting FL-PAINT image is shown in Fig. 2l. A lifetime threshold was used to separate the two targets in the FL-PAINT image into two separate images: peroxisomes in Fig. 2j and mitochondria in Fig. 2k. The average localization precision for Exchange-PAINT was 14.6 nm for the peroxisome image and 13.5 nm for the mitochondria image. The average localization precision for FL-PAINT was 20.3 nm for peroxisomes and 16.5 nm for mitochondria; the localization precision in the original FL-PAINT image was 17.6 nm. Further quantitative characterization of the images are provided in Supplementary Table 2. The mitochondria images shown in Fig. 2h, k show perfect correlation, while the peroxisomes images shown in Fig. 2g, j have visible differences. This is due to a slight *z*-axis drift that occurred between the two acquisitions as a result of buffer washing procedures performed between the acquisitions. Such *z*-axis drift that might occur in Exchange-PAINT experiment emphasizes the conceptual advantage of FL-PAINT, where multi-target imaging is done simultaneously then avoiding buffer exchange and consequent sample disturbance.

### Confocal FL-PAINT

**Fig. 3:**
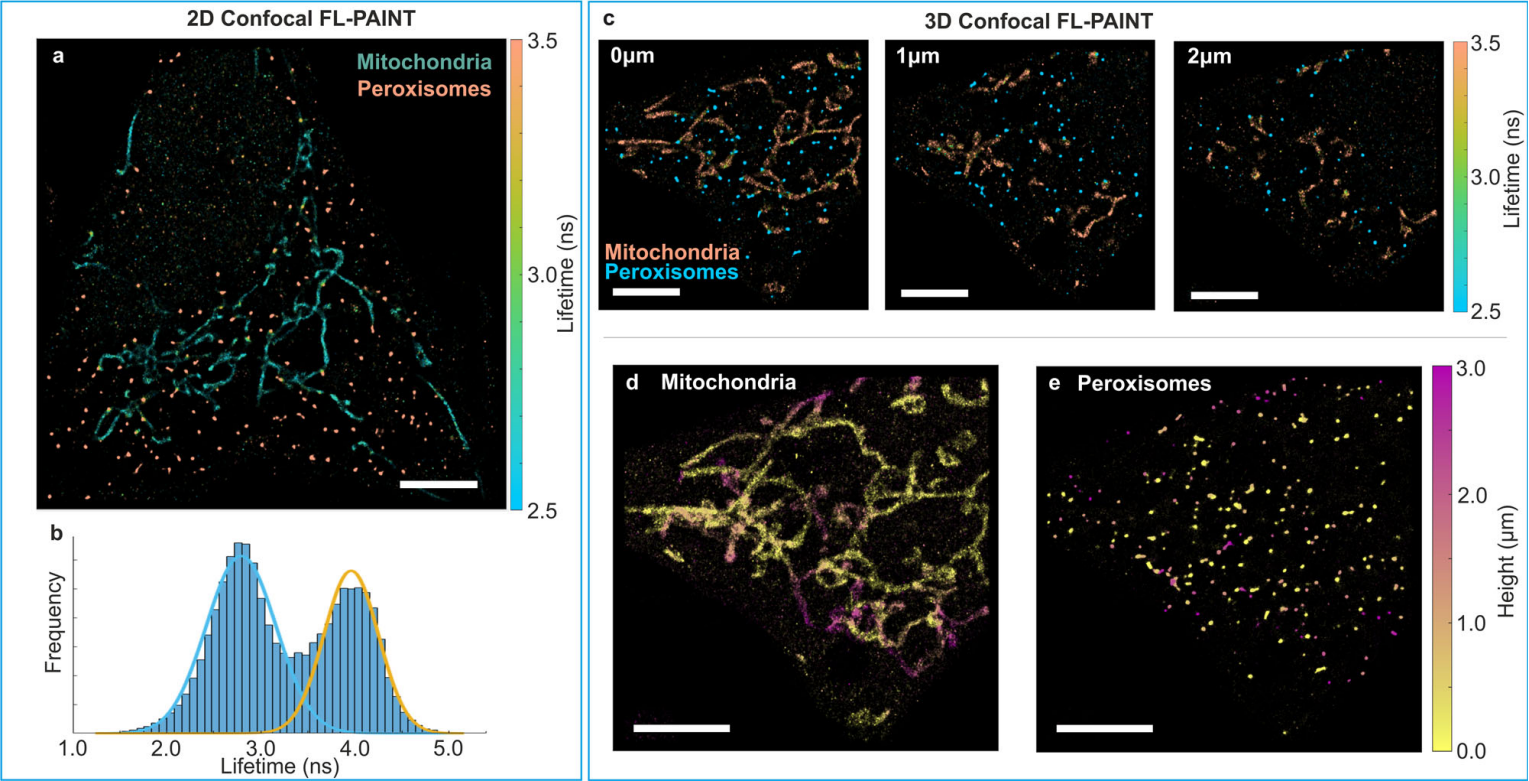
2D and 3D FL-PAINT using a CLSM. **a** 2D CLSM FL-PAINT image of a HeLa cell with two targets: peroxisomes and mitochondria, labeled with the imagers P1-Atto 550 and P3-Cy3b. **b** The corresponding lifetime histogram with two Gaussian fits for the cell shown in (**a**). Colors of fitted Gaussians correspond to the colors of the targets shown in (**a**). **c** 2D CLSM FL-PAINT images of a HeLa cell again with two targets: peroxisomes and mitochondria, but now labeled with P1-Alexa 555 and P3-Atto 550 imagers. The images correspond to focal planes at 0, 1, and 2 µm axial distance above the bottom of the cell. Z-projections of the separated images for mitochondria (**d**) and peroxisomes (**e**). Here, the color represents the height. Lifetime and height color bars are shown on the right-hand side of the images. Scale bars are 5 µm.

As an alternative to the not yet widely available LINCam lifetime camera used above, we next demonstrate the feasibility of performing FL-PAINT with a CLSM capable of TCSPC measurements^3^. As a sample, we used again HeLa cells labeled with a mixture of the two imagers P1-Atto 550 and P3-Cy3b. We used a custom-built CLSM (for detailed optical setup schematic see Supplementary Fig. 1b) to image the same sample with HeLa cells as used for wide-field FL-PAINT. Fast scanning with frame rates of 5–10 Hz was performed in order to collect a sufficient number of photons from each imager-docking binding event. Further experimental details can be found in the “Methods” section. A typical lifetime image is shown in Fig. 3a. In the corresponding lifetime histogram, two distinct peaks are visible, corresponding to the two targets, peroxisomes, and mitochondria, respectively (Fig. 3b).

One of the main benefits of CLSM is its ability to perform optical sectioning, which allows the acquisition of 3D images. We demonstrate two-target 3D FL-PAINT by imaging HeLa cells with imagers P1-Alexa 555 revealing peroxisomes and P3-Atto 550 revealing mitochondria (Fig. 3c−e). Image acquisition was performed across a 3 µm z-stack with steps of 500 nm. The resulting two-target FL-PAINT images in different planes of the z-stack are displayed in Fig. 3c. Based on the lifetime values, we sorted localization events into two subgroups corresponding to mitochondria (Fig. 3d) and peroxisomes (Fig. 3e).

The CLSM is advantageous when imaging relatively small regions of interest—20 µm × 20 µm or less. CLSM is also the method of choice for imaging fluorophores in the red and far-red spectral regions, where the LINCam’s QY is less than 5%, or if one is interested in capturing 3D images of a sample. For the cells shown in Fig. 3, the average localization precision was 10.8 nm.

### Software for FL-PAINT data analysis

Single-molecule lifetime analysis was performed using a custom-written MATLAB-based GUI program. The original version of the GUI was called TrackNTrace (TNT)^30^, while its next version, called TNT Lifetime Edition, already includes the option to perform lifetime analysis of localizations and to produce super-resolution FLIM images^4^. Here, we extended the TNT program to perform FL-PAINT data analysis. It features extended capabilities for FLIM data analysis: processing wide-field and confocal FL-PAINT data formats, advanced data filtering options, drift correction, and a data visualizer, see the “Data analysis” section for more details. The software is available via GitHub at the following link: https://github.com/scstein/TrackNTrace.

## Conclusions

In summary, we developed FL-PAINT by combining DNA-PAINT with FLIM. The technique is particularly suitable for multiplexed DNA-PAINT imaging. Due to the robustness of lifetime-based target identification, FL-PAINT is insensitive to the imager-docking binding kinetics and therefore performs exceptionally well in the dense and sticky environment inside a cell. Moreover, the sample is not disturbed during data acquisition, as fluid exchange and consequently, a dedicated microfluidic system is not required. FL-PAINT enables simultaneous imaging of multiple targets, therefore shortening the total acquisition time. We identified a combination of three bright fluorophores with similar emission spectra in the orange spectral range, but different lifetimes, which are an excellent choice for simultaneous two- and three-target FL-PAINT imaging. We implemented FL-PAINT using both wide-field FLIM (equipped with commercially available lifetime camera) and time-resolved CLSM (equipped with TCSPC electronics and fast laser scanning). Wide-field FL-PAINT enables HILO/TIRF illumination and is faster than CLSM FL-PAINT. However, the localization precision is better for CLSM-based FL-PAINT due to the higher detector quantum yield. CLSM approach is particularly advantageous for imaging small regions of interest, and it enables 3D imaging thanks to the optical sectioning capability of the technique. We emphasize that FL-PAINT uses fluorophores emitting in the same spectral range, and therefore is not affected by the chromatic aberration. We further developed a freely available data analysis software. FL-PAINT is compatible with other DNA-PAINT-based techniques of multiplexed imaging, and therefore FL-PAINT has a great potential for highly multiplexed bioimaging. Finally, FL-PAINT provides fluorescence lifetime information, making it attractive for single-molecule FRET^31–33^ and metal-induced energy transfer (MIET)^34,35^.

## Data analysis

For confocal FL-PAINT data analysis, we chose a time binning of eight scanned frames, corresponding to the time bin of 0.8 s for a scan region of 20 µm × 20 µm and 1.8 s for a scan region of 30 µm × 30 µm, at a pixel dwell time of 2.5 µs. The detection and precise sub-pixel localization of emitters were performed using a cross-correlation algorithm and pixel-integrated Gaussian MLE fitting. Molecules that were detected in only one frame were discarded. Also, localizations with a PSF width of more than 180 nm or a number of photons smaller than 100 were rejected. For lifetime determination, we discarded the first 0.1 ns after the maximum of the TCSPC histograms, and the remaining “tails” were fitted with a mono-exponential function using a maximum likelihood estimator (MLE). Only lifetime values in the range from 0.5 to 5.0 ns were taken into account. Subsequently, a drift correction was applied, and a FL-PAINT image was reconstructed.

For wide-field FL-PAINT data analysis, we chose a spatial binning of 8 pixels (corresponding to a virtual pixel size of 192 nm) and a time bin of 500 ms. Again, molecules that were detected in only one frame were discarded. Similarly, localizations with a PSF width of more than 345 nm and a number of photons smaller than 100 were rejected as well. After that, a drift correction was applied, and a super-resolution image was reconstructed.

## Methods

### Preparation of HeLa cells

HeLa cells were cultured in low glucose Dulbecco’s Modified Eagle Medium (DMEM) medium (Gibco) supplemented with 1% Pen/Strep (100 units/ml penicillin and 100 µg/ml streptomycin), 1% (w/v) glutamine, and 10% (v/v) Fetal Calf Serum in 5% CO_2_ at 37 °C. 16000 cells were seeded on eight-well chambered coverglass (Cellvis) and transfected with GFP-PTS1 (PST990), mito-BFP (gift from Gia Voeltz, Addgene plasmid #49151) and mCherry-FRB-VAPB (gift from Ralph Kehlenbach) using Effectene (Qiagen) transfection reagent according to the manufacturer’s instructions. Twenty-four hours after transfection, cells were washed with phosphate-buffered saline (PBS), fixed with 4% PFA in PBS for 30 min, and permeabilized for 10 min using 1% Triton X-100 in PBS.

### Preparation of COS-7 cells

COS-7 cells were cultured in DMEM medium supplemented with 4 mM l‐glutamine, 10% (v/v) Fetal Calf Serum (Thermo Fisher Scientific), 60 U/ml of penicillin, and 0.06 mg/ml streptomycin (Sigma‐Aldrich) in 5% CO2 at 37 °C. Prior to plating the cells, ca. 20000 cells/well were triple transfected for mitochondria, histones, and endoplasmic reticulum (ER) with 70 ng TOM70-GFP, 131 ng H2B-mTagBFP, and 131 ng GalNac-mCherry, respectively. During transfection, 2% lipofectamine 2000 was used according to the manufacturer’s instructions. After transfection, cells were plated in eight-well chambers (155411PK, Thermo Fisher Scientific), incubated for ca. 16 h, and subsequently fixed using 4% paraformaldehyde (PFA) for 30 min at room temperature. The remaining aldehydes were quenched with 0.1 M glycine in PBS for 15 min.

### Immunostaining

Cells were permeabilized and blocked using 2% bovine serum albumin (BSA) and 0.1% Triton X-100 in PBS for 30 min at room temperature. Buffer solution containing nanobodies coupled to the docking strand (50 nM) was used to stain the cells. For this purpose, we incubated cells for 1 h at room temperature with slow orbital shaking. Finally, cells were rinsed with PBS and then post-fixed with 4% PFA for 15 min at room temperature. The remaining aldehydes were quenched by 0.1 M glycine in PBS. Cells were stored in PBS at 4 °C. The unconjugated nanobodies FluoTag-Q anti-GFP, FluoTag-Q anti-RFP, and the FluoTag-Q anti-TagBFP (NanoTag Biotechnologies GmbH, Germany, Cat. No: N0301, N0401, and N0501, respectively) carry one ectopic cysteine at the C-terminus then allowing for chemical couplings via a thiol reactive compound. The docking strands sequences used for the assay were taken from Agasti et al.^22^. DNA docking strands (Biomers GmbH, Germany) were functionalized with an azide group at 5′-end. Coupling of the docking strands to the nanobodies was performed using dibenzocyclooctyne (DBCO) cross-linker^11^. FluoTag-Q anti-GFP was coupled to P1* sequence 5′-TTATACATCTA-3′, FluoTag-Q anti-RFP was coupled to P2* sequence 5′-TTATCTACATA-3′, and FluoTag-Q anti-TagBFP was coupled to P3* sequence 5′-TTTCTTCATTA-3′.

### Data acquisition

*Wide-field FL-PAINT*: Fluorescence lifetime imaging was performed on a custom-built optical setup equipped with a lifetime camera (LINCam25, Photonscore, Germany), see Supplementary Fig. 1a. The imager strands P1 5′-CTAGATGTAT-3′, P2 5′-TATGTAGATC-3′, and P3 5′-GTAATGAAGA-3′ (Eurofins Genomics, Germany) were labeled with Cy3b, Atto 550, and Alexa 555 fluorophores at the 3′ end. All imager strands were aliquoted in TE buffer (Tris 10 mM, EDTA 1 mM, pH 8.0) at a concentration of 100 µM and stored at −20 °C. Prior to the experiment, the strands were diluted to the final concentration of 0.5 nM in PBS buffer, containing 500 mM NaCl. A chamber with eight wells (155411PK, Thermo Fisher Scientific) was fixed with clips on the microscope stage. A PDMS layer was used as a chamber cover and supported the inlet tubes and one tube for suction. The slide was kept on the microscope stage for 0.5 h before acquisition to allow thermal equilibration to room temperature and to avoid subsequent mechanical drift. First, the well was rinsed twice with 500 µL PBS buffer (pH 8.0, NaCl 500 mM). Then, using an emCCD camera, suitable cells were selected for imaging, based on the presence of signal from expressed fluorescent proteins: mTagBFP, mCherry, and EGFP. Afterwards, we proceeded with FL-PAINT of the selected cell. All solutions were injected into the well by applying air pressure to the corresponding tube. A mix of imager strands P1, P2, and P3 with final concentrations of ~0.1 nM in PBS buffer were injected into a well and incubated for 5–10 min before image acquisition. The typical laser power was ~3 mW (output of optical fiber). HILO sheet illumination was used to improve the signal-to-noise ratio. In the emission path, a neutral density filter with optical density 0.3 (NE03A-A, Thorlabs) was used to adjust the photon detection rate to an optimal photon detection efficiency of the lifetime camera. The impact of photon flux attenuation on the localization precision is shown in Supplementary Figs. 5 and 6. Image magnification was set to 222×, resulting in the partitioning of the LINCam’s field of view into 512 × 512 pixels. Further single-molecule lifetime analysis was performed as described in “Data analysis” section. All experiments were done at a constant temperature of 22.0 ± 0.3 °C. This was crucial for the mechanical stability of the optical setup. Typical acquisition time per image varied between 45 and 60 min, depending on the imager concentration and brightness of the fluorophores used for imaging.

*Confocal FL-PAINT*: Fluorescence lifetime measurements were performed on a custom-built confocal setup, see Supplementary Fig. 1b. Typically, 10^4^ scan images with a virtual pixel size of 100 nm, a dwell time of 2.5 µs/pixel, and a TCSPC time resolution of 16 ps were recorded. The typical total acquisition time was around 1–1.5 h for a 20 µm × 20 µm scan region. A mix of imager strands P1 and P3 with final concentrations of 0.1 nM in PBS buffer including NaCl 500 mM were injected into the chamber and incubated for 5–10 min before image acquisition.

### Reporting summary

Further information on research design is available in the Nature Research Reporting Summary linked to this article.

## Supplementary information

Supplementary Information

Reporting Summary

## Data Availability

The data that support the findings of this study are available from the corresponding author upon reasonable request.

## Code availability

The code for FL-PAINT data analysis is available via GitHub at the following link: https://github.com/scstein/TrackNTrace.
