# Supplementary material for: Fluorescence lifetime DNA-PAINT for multiplexed super-resolution imaging of cells
Source: Commun Biol. 2022 Jan 11;5:38. doi: 10.1038/s42003-021-02976-4 (PMC8752799; doi:10.1038/s42003-021-02976-4)
Supplement: Supplementary file 1 — Supplementary Information [file 42003_2021_2976_MOESM1_ESM.pdf]

Supplementary information

Fluorescence Lifetime DNA-PAINT for Multiplexed Super-resolution Imaging of Cells

Nazar Oleksiievets[1], Yelena Sargsyan[2], Jan Christoph Thiele[1], Nikolaos Mougios[3,4], Shama Sograte-Idrissi[3,4], Oleksii Nevskyi[1], Ingo Gregor[1], Felipe Opazo[3,4,5], Sven Thoms[2,6], Jörg Enderlein[1,7,*] and Roman Tsukanov [1,*]

1. III. Institute of Physics - Biophysics, Georg August University, 37077 Göttingen, Germany.
2. Department of Child and Adolescent Health, University Medical Center Göttingen, 37073 Göttingen, Germany.
3. Institute of Neuro-and Sensory Physiology, University Medical Center Göttingen, 37073 Göttingen, Germany.
4. Center for Biostructural Imaging of Neurodegeneration (BIN), University Medical Center Göttingen, 37075 Göttingen, Germany.
5. NanoTag Biotechnologies GmbH, 37079 Göttingen, Germany.
6. Biochemistry and Molecular Medicine, Medical School, Bielefeld University, 33615 Bielefeld, Germany.
7. Cluster of Excellence “Multiscale Bioimaging: from Molecular Machines to Networks of Excitable Cells” (MBExC), Georg August University, Göttingen, Germany.

Corresponding authors: jenderl@gwdg.de (Jörg Enderlein), rtsukan@gwdg.de (Roman Tsukanov)

Supplementary Notes

Supplementary Note 1

Wide-field FLIM optical setup

Excitation was done using a pulsed super-continuum white light laser (Fianium WhiteLaser SC450, NKT Photonics) with a pulse repetition rate of 20 MHz. A custom photodiode (PD) was employed to optically trigger the TCSPC-based camera. A white light laser source in combination with clean-up filters (ZET 640/10, Chroma; BrightLine HC 563/9, Semrock; ZET 488/10, Chroma) was used to excite fluorophores with different excitation spectra (blue, green and red). Neutral density filters (NE10A-A, NE20A-A, Thorlabs) and a variable neutral density filter (ND) (NDC-50C-4-A, Thorlabs) were used for adjusting the laser excitation power. The laser beam was coupled into a single-mode optical fiber (P1-460B-FC-2, Thorlabs) with a typical coupling efficiency of 50%. After the fiber, the beam was collimated and expanded by a factor of 3.6X using telescope lenses. The beam was focused onto the back focal plane of the TIRF objective (UAPON 100× oil, 1.49 NA, Olympus) using an achromatic lens (f = 200 mm, AC508-200-A-ML, Thorlabs). Switching between direct, HILO, and Total Internal Reflection (TIR) illumination was achieved by shifting the beam laterally with respect to the objective lens using a translation stage (LNR50M, Thorlabs). Fluorescence emission light was collected using the same objective lens. Samples were placed onto a XY translation stage (M-406, Newport). An independent one-dimensional translation stage (LNR25/M, Thorlabs) was equipped with a differential micrometer screw (DRV3, Thorlabs) for axially moving the objective lens for focusing. Collected fluorescence light was spectrally decoupled from scattered excitation laser light using a multi-band dichroic mirror (Di03 R405/488/532/635, Semrock) and band-pass filters (BrightLine HC 692/40, Semrock; BrightLine HC 615/45, Semrock; BrightLine FF 536/40, Semrock). A tube lens (AC254–200-A-ML, Thorlabs) focused the light on an adjustable slit aperture (SP60, OWIS). The latter was employed to select a region of interest within the field of view. Two lenses (AC254-100-A and AC508–150-A-ML, Thorlabs) were used to focus the light onto an emCCD camera (iXon Ultra 897, Andor). Similarly, lens L5 (AC508-250-A-MC, Thorlabs) re-imaged the light onto the lifetime camera (LINCam25, Photonscore). Switching between the two cameras was attained by sliding a dielectric mirror (BB1-E02, Thorlabs) into and out of the optical path (MB). A schematic of the setup is shown in Supplementary Figure 1a.

Confocal optical setup for FL-PAINT imaging

Fluorescence lifetime measurements were performed with a custom-built confocal setup. For excitation, a white light laser (Fianium WhiteLase SC450, NKT Photonics) was used. The laser beam was coupled into a single-mode fiber (PMC-460Si-3.0-NA012–3APC-150-P, Schäfter + Kirchhoff) with a fiber-coupler (60SMS-1-4-RGBV-11-47, Schäfter + Kirchhoff). After the fiber, the output beam was collimated by an air objective (UPlanSApo 10× /0.40 NA, Olympus). After passing thought a clean-up filter (BrightLine HC 563/9, Semrock), a quad-band dichroic mirror (ZT405/488/561/640rpc, Chroma) was used to reflect the excitation light towards the microscope. The excitation beam was directed into a laser scanning system (FLIMbee, PicoQuant) and then into one of the side ports of the microscope (IX73, Olympus). The three galvo mirrors in the scanning system are deflecting the beam, while preserving the lateral beam position in the back focal plane of the objective (UAPON 100× /1.49 NA oil, Olympus). The sample position was adjusted using a manual XY stage (Olympus) and a z-piezo stage (Nano-ZL100, MadCityLabs). Fluorescence light was collected by the same objective and de-scanned in the scanning system. Afterwards, an achromatic lens L2 (TTL180-A, Thorlabs) was used to focus the beam onto a 100 µm pinhole (P100S, Thorlabs). Excitation laser light was blocked by a long-pass filter LP (647 LP Edge Basic, Semrock). Next, the emission light was collimated by a 100 mm lens L3. A band-pass filter BP (BrightLine HC 615/45) was used to reject scattered excitation light. Finally,

the emission light was focused onto a single-photon avalanche photo-diode (SPAD) (SPCM-AQRH, Excelitas) using an achromatic lens L4 (AC254–030-A-ML, Thorlabs). The output signal of the SPAD was recorded by a TCSPC system (HydraHarp 400, PicoQuant), which in turn was synchronized with the triggering signal from the excitation laser. Measurements were controlled with a dedicated software (SymPhoTime 64, PicoQuant), which controlled both the TCSPC and the scanner systems. A schematic of the setup is shown in Supplementary Figure 1b.

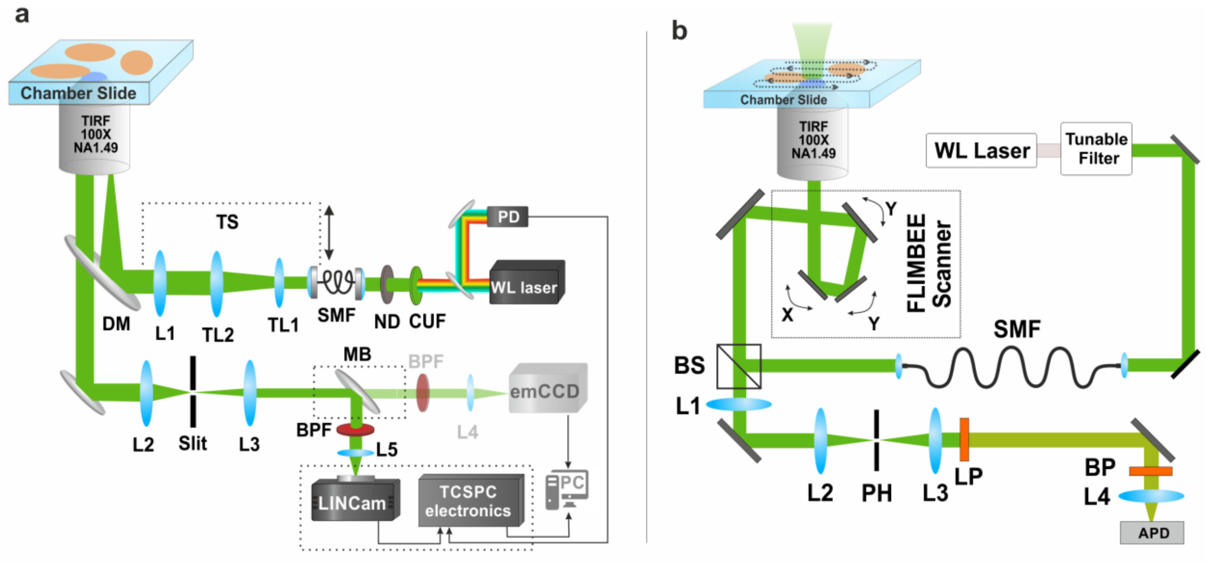

Supplementary Figure 1. Experimental FLIM techniques compatible with FL-PAINT. (a) Conventional wide-field microscope equipped with a lifetime camera. (b) Confocal-based laser scanning microscope equipped with TCSPC electronics and fast scanner.

Supplementary Note 2

Lifetimes of fluorophores conjugated to imager strands

Lifetimes of fluorophores are affected by the presence of single-stranded DNA in a sequence-dependent manner. In Supplementary Table 1, we provide lifetime values of all fluorophore-imager combinations measured in cells in a single-target measurement. Wide-field FL-PAINT imaging was employed.

Supplementary Table 1. Average lifetime values and average number of photons per localization (single binding event) of DNA-fluorophore complexes measured with the LINCam.

Imager strand	Fluorophore	Lifetime (ns)	Number of photons/localization
P1	Alexa 555	1.80 ± 0.22	511
P1	Atto 550	3.44 ± 0.26	1318
P2	Alexa 555	1.58 ± 0.11	751
P2	Atto 550	3.81 ± 0.31	1402
P3	Cy3b	2.82 ± 0.16	1117
P3	Atto 550	3.77 ± 0.30	1778

Supplementary Note 3

Quantitative characterization of FL-PAINT images

In Supplementary Table 2, we provide the following information for all FL-PAINT images presented. This includes fluorescence lifetimes of different dye-imager complexes measured in a mix, average localization precision, average resolution, minimum resolution, and number of localization events.

Supplementary Table 2. Quantitative characterization of FL-PAINT images: fluorescence lifetime of imager-fluorophore complexes, average localization precision, average resolution, minimum resolution, and number of localization events.

Figure	Cell	Optical setup	Imager	Lifetime (ns)	ND filter (OD)	Average localization precision (nm)	Average resolution (nm)	Min resolution (nm)	Number of localization events
Fig. 1d	HeLa	Wide-field	P1-Alexa 555<br>P3-Atto 550	1.85 ± 0.26<br>3.77 ± 0.30	0.3	17.6	66.4	21.3	215983
Fig. 2a	HeLa	Wide-field	P1-Alexa 555<br>P3-Cy3b	1.92 ± 0.18<br>2.51 ± 0.21	0.3	21.2	64.4	18.7	218250
Fig. 2c	HeLa	Wide-field	P1-Atto 550<br>P3- Cy3b	3.70 ± 0.28<br>2.83 ± 0.18	0.6	20.8	63.3	21.0	292615
Fig. 2e	HeLa	Wide-field	P1-Alexa 555<br>P2-Atto550<br>P3- Cy3b	2.29 ± 0.20<br>3.42 ± 0.26<br>2.80 ± 0.15	0.3	22.9	74.7	21.4	213328
Fig. 2j	HeLa	Wide-field	P1-Alexa 555	1.65 ± 0.26	0.3	20.3	48.0	17.1	671215
Fig. 2k	HeLa	Wide-field	P3-Atto 550	3.71 ± 0.24	0.3	16.5	53.7	16.8	1462697
Fig. 3a	HeLa	CLSM	P1-Atto 550<br>P3- Cy3b	3.97 ± 0.38<br>2.80 ± 0.30	-	10.9	62.5	21.3	934528
Fig. 3c	HeLa	CLSM	P1- Alexa 555<br>P3-Atto 550	1.88 ± 0.23<br>3.40 ± 0.59	-	12.1	60.8	18.5	648760
Fig. S3a	COS-7	Wide-field	P1-Atto 550<br>P3- Cy3b	3.76 ± 0.27<br>2.92 ± 0.19	0.6	23.5	65.9	18.9	894455
Fig. S3c	COS-7	Wide-field	P1-Atto 550<br>P3- Cy3b	3.79 ± 0.23<br>2.87 ± 0.18	0.6	17.5	55.1	16.3	633505
Fig. S3e	HeLa	Wide-field	P1-Atto 550<br>P3- Cy3b	3.71 ± 0.22<br>2.91 ± 0.16	0.6	19.9	54.9	18.7	511953
Fig. S3g	HeLa	Wide-field	P1-Alexa 555<br>P2-Atto 550<br>P3- Cy3b	2.26 ± 0.26<br>2.75 ± 0.13<br>3.36 ± 0.28	-	17.5	52.8	17.6	315341
Fig. S3i	HeLa	CLSM	P1-Atto 550<br>P3- Cy3b	3.94 ± 0.30<br>2.79 ± 0.33	-	10.1	75.5	27.7	687562
Fig. S3k	HeLa	CLSM	P1-Atto 550<br>P3- Cy3b	4.00 ± 0.32<br>2.87 ± 0.32	-	10.4	96.9	21.1	491615

Supplementary Note 4

Comparison between wide-field diffraction-limited and FL-PAINT images

In our investigation, targets of interest were encoded with fluorescent proteins and then labelled with nanobody-docking complexes for FL-PAINT. This enabled validation of nanobodies labeling specificity by a comparison between diffraction-limited images of, for example, peroxisomes-mTagBFP and mitochondria-EGFP, and DNA-PAINT images of the same targets. We separated targets from an FL-PAINT image of the cell by using a lifetime threshold, see Supplementary Figure 2. Only a zoom-in of a cellular sub-region is shown. The full cell image is shown in Figure 1d in the main text.

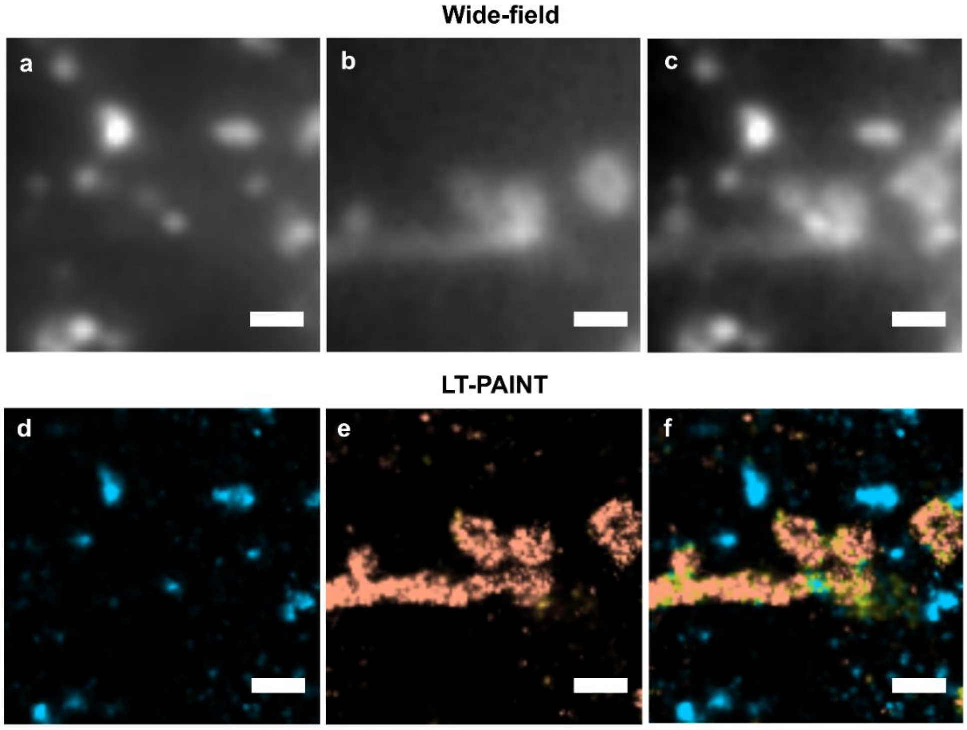

Supplementary Figure 2: (a-c) Diffraction-limited wide-field images of individual targets: peroxisomes-mTagBFP (a) and mitochondria-EGFP (b). (c) Merged wide-field image. Lifetime values were used to separate the targets from FL-PAINT image into two images: peroxisomes (d) and mitochondria (e). (f) Two-target FL-PAINT image of both targets. Scale bars are 1 µm.

Supplementary Note 5

Exchange-PAINT imaging

Exchange-PAINT experiments were performed using the same custom-built optical setup as used for FL-PAINT but with a different emission path equipped with a conventional emCCD camera. Injection and removal of solutions was done using our custom-built microfluidics setup, designed and constructed especially for Exchange-PAINT[1]. First, P1 imager strand (stock concentration 1 µM) was diluted in PBS buffer including 500 mM NaCl (500 µL volume) to the concentration of 0.5 nM and injected into the chamber followed by 5 min incubation prior to image acquisition. A typical DNA-PAINT movie included 20000 frames, which corresponds to a total acquisition time of 30 min. The following settings for the emCCD camera were used: exposure time 100 ms, pre-amplifier gain 3.0, EM gain 100. The first round of image acquisition was followed by removal of P1 imager from the chamber and by an extensive washing of the chamber with the imaging buffer (4–6 times the chamber volume, in total about 2 mL buffer within 5 min). After washing, the next imager P3 was introduced.

Same procedure was repeated also for the imager P3. Recorded images were analyzed with ImageJ plugin ThunderSTORM[2] for determining the positions of single emitters and reconstructing a super-resolution image, see main text Figure 2g-h for each separate targets and Figure 2i for 2-channel overlay. In all the experiments, the same parameters were used for emitter localization and subsequent super-resolution image reconstruction.

Supplementary Note 6

Target crosstalk estimation

In this section, we discuss a quantitative estimation of target crosstalk for the cell images shown in the main text. For this purpose, we fit the lifetime distribution histograms with two (or three) Gaussians and set lifetime threshold values to separate between the different targets. We determine the total crosstalk for two or three targets as the total number of localizations in the distribution beyond a threshold (wrong attribution), divided by the total number of localizations within the threshold bounds (correct attribution), see Supplementary Figure 3.

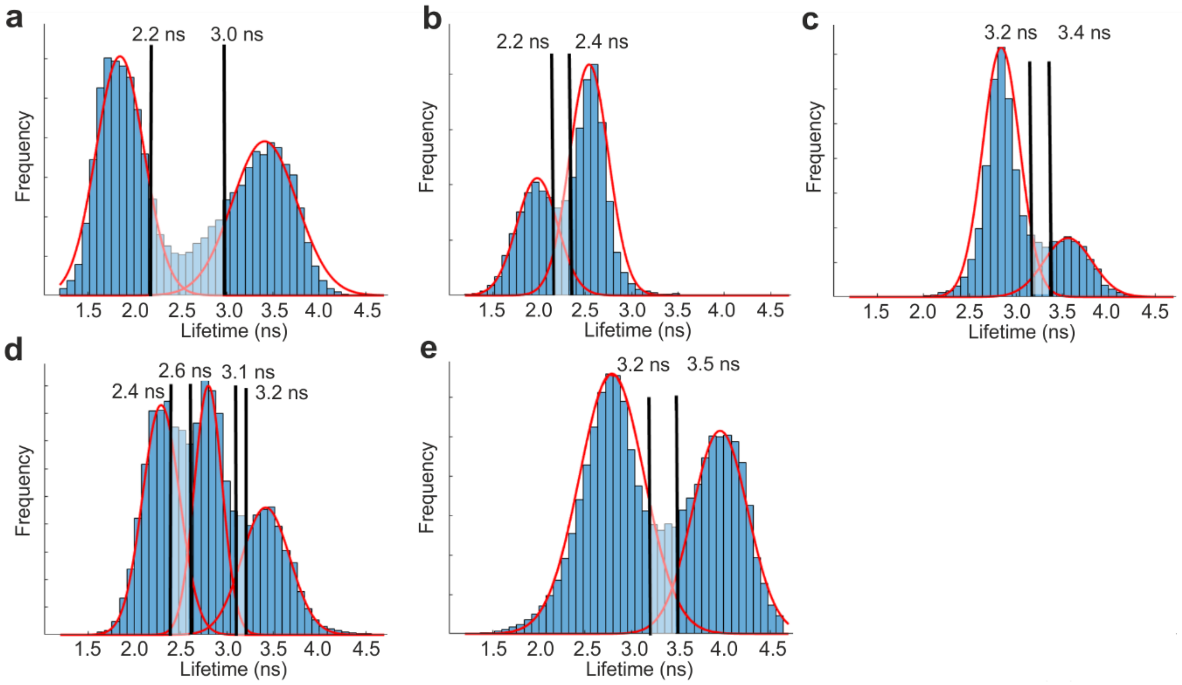

Supplementary Figure 3: Target crosstalk evaluation in FL-PAINT. Lifetime distribution histograms with the fit of two Gaussians (red curves) are shown. Crosstalk is calculated based on lifetime threshold values (black solid lines). To decrease the crosstalk, localizations between two lifetime threshold values were excluded (histogram regions marked in light blue color). Analysis of HeLa cell images from main text: (a) cell image in Figure 1 – two-target total cross-talk 0.1%; (b) cell image in Figure 2a – two-target total crosstalk 4.4%; (c) cell image in Figure 2c – two-target total crosstalk 2.2%; (d) cell image in Figure 2e – three-target total crosstalk 5.7%; (e) cell image in Figure 3a – two-target total crosstalk 1.6%.

Supplementary Note 7

Resolution estimation for FL-PAINT images

To quantitatively estimate the image resolution, Fourier Ring Correlation (FRC) algorithm was used[3]. The values for average and minimal resolutions can be found in Supplementary Table 2. The resolution maps were created using Super-resolution quantitative image rating and reporting of error locations (SQUIRREL)[4] plugin for ImageJ. The exemplary resolution map for the cell shown in Figure 1 in the main text are shown in Supplementary Figure 4.

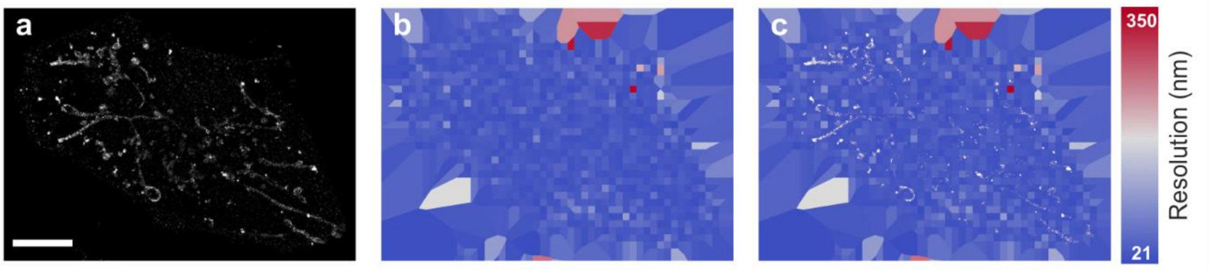

Supplementary Figure 4: Resolution map for FL-PAINT image. (a) Two-target super-resolution FL-PAINT image of Hella cell from Figure 1 in the main text. (b) Fourier Ring Correlation (FRC) resolution map obtained by NanoJ-SQUIRREL, the plugin for ImageJ. (c) FRC map overlaid with the super-resolution image in (a). The color bar on the right-hand side encodes the FRC resolution. Scale bar is 5 µm.

Supplementary Note 8

Zoom-ins of peroxisomes

To further demonstrate the resolution of images obtained using FL-PAINT, we show intensity profile along cross-sections of different peroxisomes. Peroxisomes dimensions are in range of 0.1-1 µm in diameter[5,6]. In Supplementary Figure 5a and b, intensity profiles along the cross-section of peroxisomes are shown and FWHM is calculated for each peroxisome. In addition, peroxisomal reticulum structures were observed during the experiment, see images and corresponding cross-sections on Supplementary Figure 5c-e.

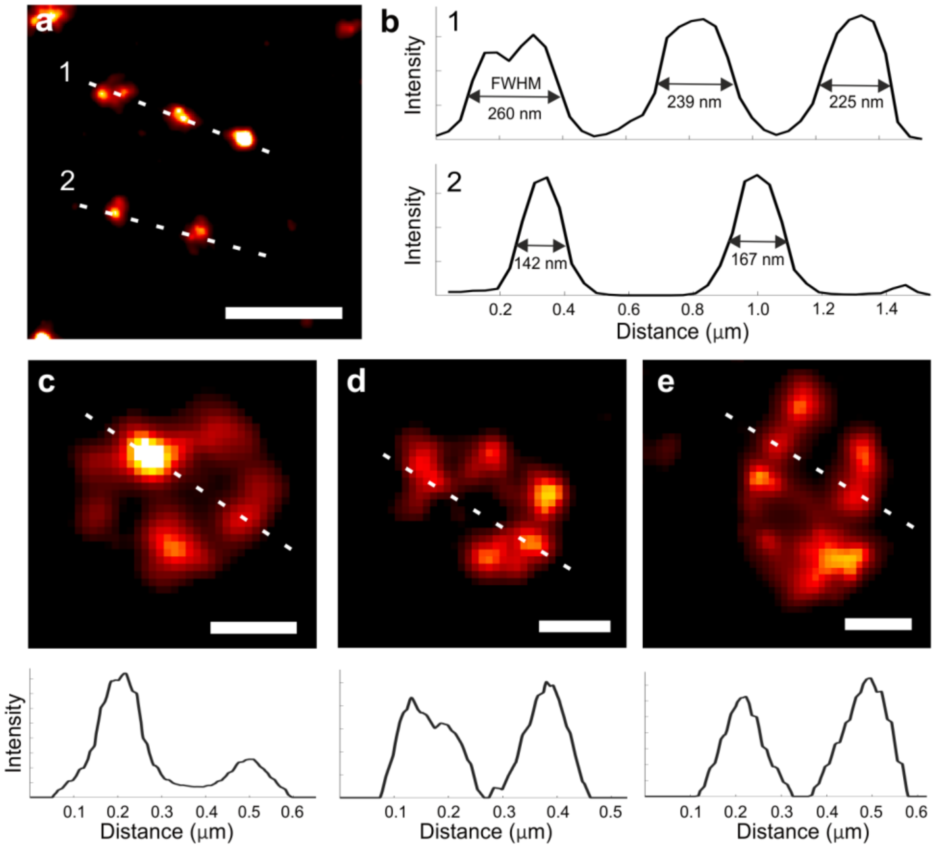

Supplementary Figure 5: Zoom-ins of peroxisomes. (a) Super-resolution FL-PAINT image of peroxisomes in HeLa cell shown Figure 2c in the main text. (b) Intensity profiles of cross sections 1 and 2, as depicted by white dashed lines in (a). Scale bar is 1 µm. (c-e) Super-resolution FL-PAINT images and cross sections of peroxisomes (peroxisomal reticulum) from Figures 1d and 2c in the main text. Scale bars are 0.2 µm.

Supplementary Note 9

Additional FL-PAINT images with different imager-fluorophore combinations

We explored the potential of FL-PAINT by testing different imager-fluorophore combinations in COS-7 cells with P1-Atto 550 and P3-Cy3b (Supplementary Figure 6a-d), HeLa cell with P1-Atto 550 and P3-Cy3b (Supplementary Figure 6e-f) and P1-Alexa 555, P2-Atto 550, and P3-Cy3b (Supplementary Figure 6g-h). In addition, we performed CLSM FL-PAINT in HeLa cells with P1-Atto 550 and P3-Cy3b (Supplementary Figure 6i-l).

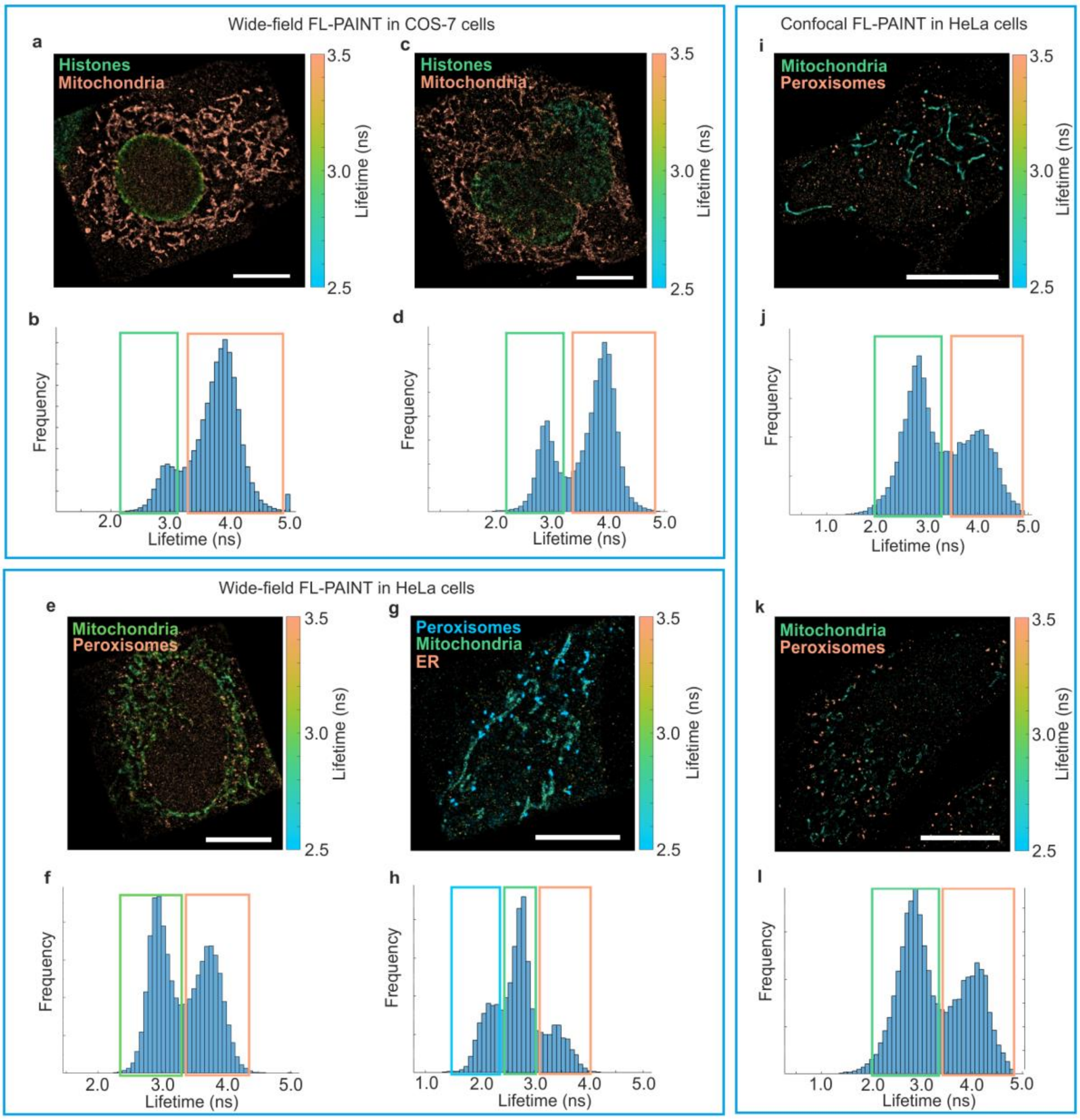

Supplementary Figure 6. FL-PAINT images of HeLa and COS-7 cells. (a,c) Two-target FL-PAINT images of COS-7 cell: mitochondria and histones labeled with imagers P1-Atto 550 and P3-Cy3b. (b,d) Lifetime histograms for COS-7 cells shown in (a,c). The two peaks in the lifetime histogram correspond to the two targets: mitochondria and histones. (e) Two-target FL-PAINT image of HeLa cell: peroxisomes and mitochondria labeled with imagers P1-Atto 550 and P3-Cy3b. (g) Three-target FL-PAINT image of HeLa cell: peroxisomes, endoplasmic reticulum, and mitochondria labeled with imagers P1-Alexa 555, P2-Atto 550, and P3-Cy3b. (f, h) Lifetime histograms for HeLa cells shown in (e, g). The peaks in the lifetime histogram correspond to the two or three targets: peroxisomes, mitochondria, and endoplasmic reticulum. (i, k) Two-target CLSM FL-PAINT images of HeLa cell: peroxisomes and mitochondria labeled with imagers P1-Atto 550 and P3-Cy3b. (j, l) Lifetime histograms for HeLa cells shown in (i, k). The peaks in the lifetime histogram correspond to the two targets: peroxisomes and mitochondria. Scale bars are 10 µm.

Supplementary Note 10

Bayesian pattern matching algorithm for targets identification

Our Bayesian pattern matching analysis assumes equal prior probabilities for all species. The analysis was performed as described elsewhere[7]. Using the algorithm, we analyzed cell shown in Figure 2 in the main text, for the comprehensive comparison of targets separation quality. Briefly, reference TCSPC curves were measured on single-imager samples, see Supplementary Figure 7a. In order to obtain a cumulative TCSPC curve, photons from filtered localization events were summed up and normalized. Localizations with more than 100 photons and a PSF width of 180 nm or smaller were taken into account. Multi-target FL-PAINT images were processed by estimating, for each localization, the probabilities that the associated TCSPC histogram was generated by each of the possible species, using the prior measured reference TCSPC curves. Supplementary Figure 7b shows histograms of the lifetime distribution for localization events and the individual species histograms obtained with the Bayesian pattern-matching algorithm. Such classification leads to high quality targets separation, as shown in Supplementary Figure 7c-e.

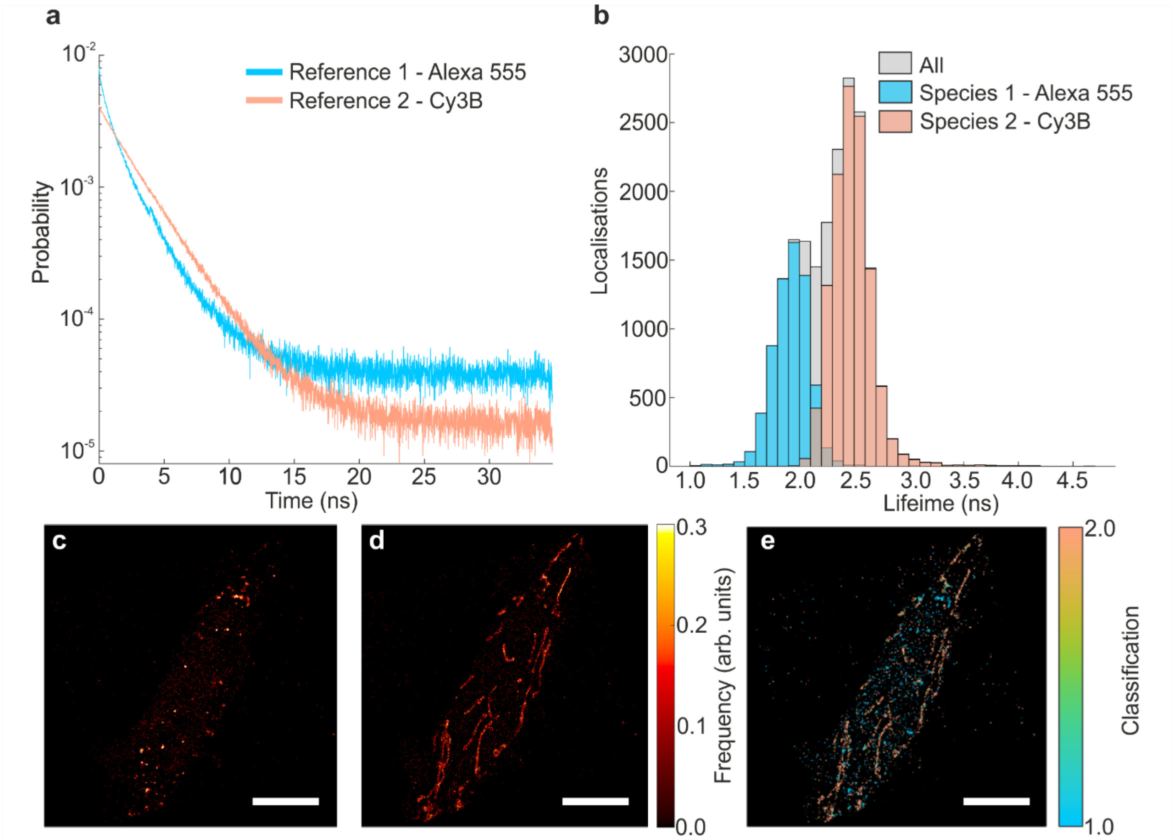

Supplementary Figure 7. Bayesian pattern matching analysis. (a) Reference TCSPC histograms measured on samples with only one imager strand (b) Lifetime distribution histograms obtained from individual single-molecule localizations for peroxisomes labeled with Alexa 555 and mitochondria labeled with Cy3b. (c-d) FL-PAINT images of localizations classified as (c) Alexa 555 and (d) Cy3b. All images share the same intensity scale, which is proportional to the local number of localizations. (e) Super-resolved dual-channel image obtained by Bayesian pattern matching analysis. Scale bar is 10 µm.

Supplementary Note 11

Lifetime camera (LINCam) photon detection efficiency

LINCam system uses a structured charge-dividing anode to acquire a photon position. Simultaneously, the system employs the signal from the microchannel plate (MCP)[8] to acquire arrival time of the photon. The recorded count-rate (e.g., the rate of useful events) is lower than the MCP count-rate. This difference between the two count-rates is due to the events selection criteria: (1) multiple events occurring within system dead time interval are rejected and (2) photons that fall outside the time-to-amplitude converter (TAC) registration time window are discarded. Knowing both the recorded and MCP count-rates, we built the camera photon detection efficiency curve (see Supplementary Figure 8a) and spotted the count-rate with minimal number of unregistered photons (around 300-350 kHz, see Supplementary Figure 8b). The curves were obtained by recording the count-rates on the camera anode and MCP at different excitation laser power, which was decreased from 5 mW to 1 mW using a continuously variable neutral density filter. As a sample, we used a solution with high concentration of imager labeled with Cy3b fluorophore, without any specific target (blank surface).

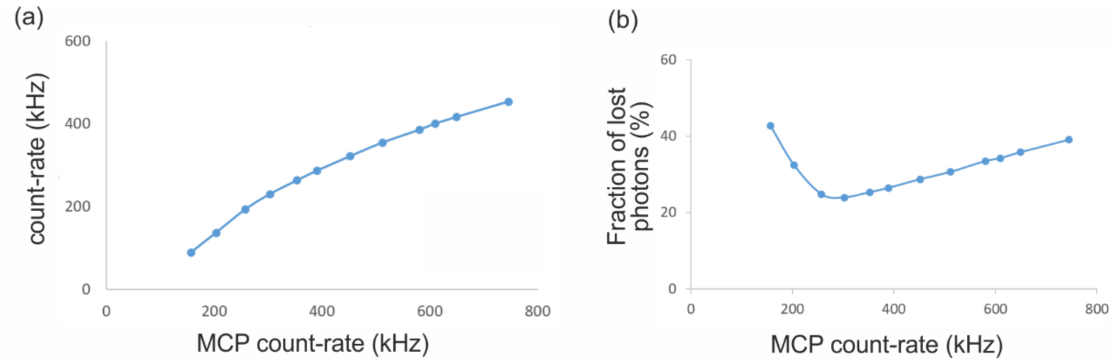

Supplementary Figure 8: LINCam lifetime camera photon detection efficiency at different photon count-rates. (a) Photon count-rate as a function of MCP photon count-rate. (b) Fraction of unregistered photons as a function of MCP photon count-rate.

Supplementary Note 12

Lifetime camera localization accuracy at reduced photon count-rates

To evaluate the impact of reduced emission photons flux on the imaging parameters, we performed wide-field FL-PAINT imaging of HeLa cell with labelled mitochondria. We kept the excitation laser power constant, and adjusted the photon flux reaching the LINCam by introducing neutral density (ND) filters with different optical densities (OD) into the detection path of optical setup. The filters ODs were 0 (no filter), 0.3, 0.6 and 1, resulting in a transmission of 100%, 50%, 25% and 10%, respectively. The same region of interest was imaged, see Supplementary Figure 9. Imager strand P3-Cy3b (concentration 0.5 nM) was used for the measurement. Each FL-PAINT dataset included 13500 frames with 200 ms time bins, which translates into total measurement time of 45 minutes. Imaging parameters were estimated based on a pixel-integrated 2D Gaussian MLE fit of the molecules PSF. For each localization, all subsequent frames in which the localization was detected were summed before fitting the PSF. The background level was determined as the offset parameter in a Gaussian fitted to each localization. Number of photons per localization, background and localization precision for attenuated emission photon flux are shown in Supplementary Figure 10.

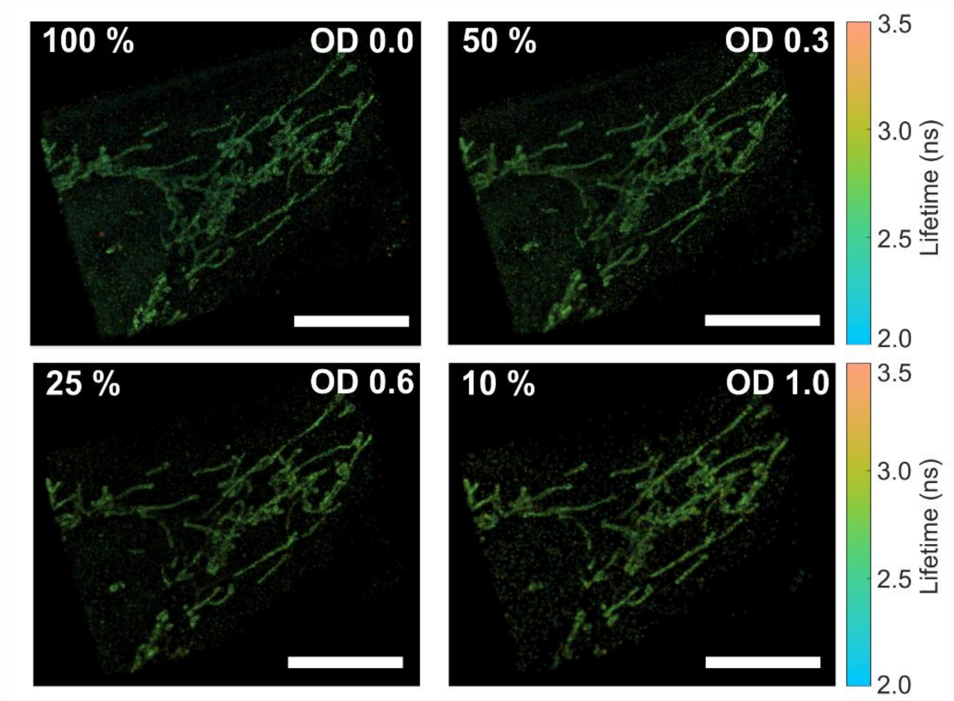

Supplementary Figure 9. Effect of emission photon detection count-rate on lifetime accuracy and localization precision. The same cell was imaged with several neutral density filters with different transmission ratio (the number in percent, as compared to an unrestricted photon flux reaching the detector) placed into the emission path of the optical setup. Lifetime color bars are shown on the right-hand side of the images. The neutral density filters transmission and OD are shown in the upper part of images. Scale bar is 10 µm.

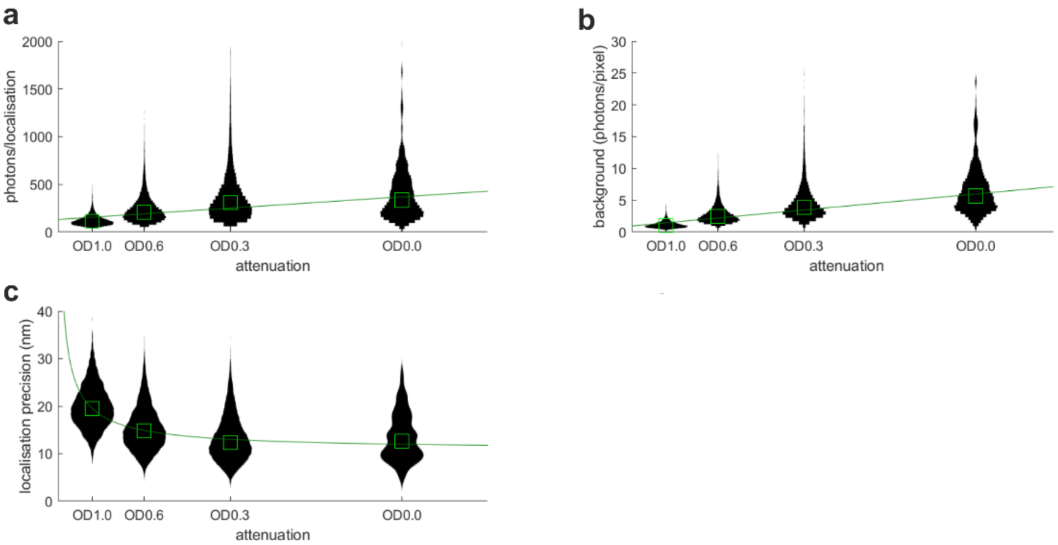

Supplementary Figure 10. Imaging parameters for attenuated emission photon flux: (a) number of photons per localization after the background subtraction; (b) background level in photons per pixel; (c) localization precision for a Gaussian MLE fit of the PSF calculated according to Rieger and Stallinga[9]. The green squares represent the median values of the data. Green lines are the linear dependency in (a) and (b) and an expected theoretical dependency calculated as 1/square root (number of photons) in (c).

Supplementary References

1 Sograte-Idrissi, S. *et al.* Nanobody Detection of Standard Fluorescent Proteins Enables Multi-Target DNA-PAINT with High Resolution and Minimal Displacement Errors. *Cells* **8**, doi:10.3390/cells8010048 (2019).

2 Ovesný, M., Křížek, P., Borkovec, J., Švindrych, Z. & Hagen, G. M. ThunderSTORM: a comprehensive ImageJ plug-in for PALM and STORM data analysis and super-resolution imaging. *Bioinformatics* **30**, 2389-2390, doi:10.1093/bioinformatics/btu202 (2014).

3 Nieuwenhuizen, R. P. J. *et al.* Measuring image resolution in optical nanoscopy. *Nature Methods* **10**, 557-562, doi:10.1038/nmeth.2448 (2013).

4 Culley, S. *et al.* Quantitative mapping and minimization of super-resolution optical imaging artifacts. *Nature Methods* **15**, 263-266, doi:10.1038/nmeth.4605 (2018).

5 Smith, J. J. & Aitchison, J. D. Peroxisomes take shape. *Nature Reviews Molecular Cell Biology* **14**, 803-817, doi:10.1038/nrm3700 (2013).

6 Soliman, K., Göttfert, F., Rosewich, H., Thoms, S. & Gärtner, J. Super-resolution imaging reveals the sub-diffraction phenotype of Zellweger Syndrome ghosts and wild-type peroxisomes. *Scientific Reports* **8**, 7809, doi:10.1038/s41598-018-24119-2 (2018).

7 Thiele, J. C. *et al.* Confocal Fluorescence-Lifetime Single-Molecule Localization Microscopy. *ACS Nano* **14**, 14190-14200, doi:10.1021/acsnano.0c07322 (2020).

8 Prokazov, Y., Turbin, E., Weber, A., Hartig, R. & Zuschratter, W. Position sensitive detector for fluorescence lifetime imaging. *Journal of Instrumentation* **9**, C12015-C12015, doi:10.1088/1748-0221/9/12/c12015 (2014).

9 Rieger, B. & Stallinga, S. The Lateral and Axial Localization Uncertainty in Super-Resolution Light Microscopy. *ChemPhysChem* **15**, 664-670, doi:https://doi.org/10.1002/cphc.201300711 (2014).
